# Fluoride exposure and blood cell markers of inflammation in children and adolescents in the United States: NHANES, 2013–2016

**DOI:** 10.1186/s12940-022-00911-6

**Published:** 2022-10-27

**Authors:** Pamela Den Besten, Christine R. Wells, Dawud Abduweli Uyghurturk

**Affiliations:** 1 Department of Orofacial Sciences, School of Dentistry, University of California, San Francisco, CA USA; 2 Statistical Methods and Data Analytics, UCLA Office of Advanced Research Computing, Los Angeles, CA USA

**Keywords:** NHANES, Fluoride, Inflammation, Blood cells count, CBC, White blood cells

## Abstract

**Background:**

Ingestion of fluoride in drinking water has been shown to result in increased cellular markers of inflammation in rodent models. However, the approximately 5–10 × increase in water fluoride concentrations required in rat and mouse models to obtain plasma fluoride concentrations similar to those found in humans has made relevant comparisons of animal to human studies difficult to assess. As an increased white blood cell count (WBC) is a marker of inflammation in humans, we used available NHANES survey data to assess the associations between plasma fluoride levels in the U.S. and blood cell counts children and adolescents.

**Methods:**

Multiple linear regressions were done to determine the association of blood cell counts and plasma fluoride in publicly available NHANES survey data from the 2013–2014 and 2015–2016 cycles. Plasma fluoride concentration measurements were available only for children aged 6 to 19, inclusive, and therefore this subpopulation was used for all analyses. Covariate predictors along with plasma fluoride were age, ethnicity, gender, and Body Mass Index (BMI).

**Results:**

Plasma fluoride was significantly positively associated with water fluoride, total WBC count, segmented neutrophils, and monocytes, and negatively associated with red blood cell count when adjusted for age, gender and BMI.

**Conclusion:**

Our finding that neutrophils and monocytes are associated with higher plasma fluoride in U.S. children and adolescents is consistent with animal data showing fluoride related effects of increased inflammation. These findings suggest the importance of further studies to assess potential mechanisms that are involved in absorption and filtration of ingested fluoride, particularly in tissues and organs such as the small intestine, liver and kidney.

## Background

Fluoride is a highly electronegative anion which, when present in saliva or other topical dental products, enhances the precipitation of calcium phosphates on the tooth enamel surface [1]. The observation that naturally fluoridated water was associated with reduced dental decay [2] lead the U.S. Public Health Service (PHS) to recommend that 1 ppm fluoride be added to the drinking water as a public health measure to prevent dental caries. In 2015, these recommendations were revised to lower the recommended concentration of fluoride in drinking water to 0.07 ppm [3]. Current estimates, posted by the U.S. Centers for Disease Control and Prevention (CDC), are that 73% of community water systems in the U.S. provide fluoridated water, and 63% of the U.S. population receives fluoridated water (https://www.cdc.gov/fluoridation/statistics/2018stats.htm).

When ingested, fluoride is first partially absorbed (approximately 25%) through the stomach in the form of hydrofluoric acid, and most of the remainder is absorbed in the small intestine, independent of pH [4, 5]. The absorbed fluoride is then filtered by the liver and kidney to achieve the final plasma fluoride concentration. In animals, fluoride in drinking water is associated with inflammation of the kidneys [6] and the small intestine [7–9], shown by increased expression of the inflammatory cytokines TNF-α and IL-1β, and by NF-κB protein.

However, the higher fluoride intake in rodents necessary to obtain plasma fluoride levels similar to those found in humans has complicated the interpretation of fluoride effects using rodent model systems. These differences may be due to anatomical differences [10–12] that affect fluoride clearance or other genetic differences, which result in the approximately 5 to 10 times lower plasma fluoride relative to water fluoride concentration in rats or mice as compared to humans. To assess the relevance of fluoride-related increases in inflammation in rats to findings in humans, we utilized data from the National Health and Nutrition Examination Survey (NHANES). Water and plasma fluoride concentrations were available for children aged 6–19 in NHANES surveys collected during the 2013–2014 and 2015–2016 cycles, and therefore we focused our analysis on fluoride-mediated inflammation in these populations.

Inflammation (both acute and chronic) is related to elevated inflammatory cytokines, which recruit and increased circulating white blood cells (WBC) [13]. WBC counts in NHANES data have been shown to be associated with factors that cause chronic inflammation such an increased Dietary Inflammatory Index (DII)[13] and obesity in children [14]. We therefore used available CBC (complete blood count) data to test the hypothesis that fluoride exposure, as indicated by plasma fluoride concentrations, is associated with increased WBC count as a biomarker of inflammation.

## Materials and methods

Water and plasma fluoride concentrations for both the 2013–2014 and 2015–2016 cycles of the NHANES survey were measured by the same laboratory at the College of Dental Medicine, Georgia Regents University, Augusta, GA. Water was collected from all households with survey participants aged birth to 19 years, and fluoride concentrations were measured in duplicate using a fluoride selective electrode (for details see https://wwwn.cdc.gov/Nchs/Nhanes/2013-2014/FLDEW_H.htm), with a lower limit of detection (LLOD) of 0.1 mg/L. Plasma fluoride concentrations were measured in duplicate using an ion-specific electrode following hexamethyldisiloxane (HMDS) diffusion (see https://wwwn.cdc.gov/Nchs/Nhanes/2013-2014/FLDEP_H.htm), with a lower limit of detection of 0.25 nmol. Subjects with water and plasma fluoride at or above the detection limit were included for analysis.

Sampling weights and variance correction variables were used when analyzing the combined 2013–2014 and 2015–2016 NHANES survey data to account for the NHANES survey design as recommended by the National Center for Health Statistics (NCHS). The subpopulation was defined as those aged 6 to 19 years, inclusive, whose fluoride plasma comment code was 0 (at or above the detection limit), and whose plasma fluoride value was less than or equal to five micromolar. We set the upper limit at 5 micromolar fluoride to preclude the possible influence of outlier values on our analysis. Values as high as 5 micromolar fluoride were found in serum of healthy women residing in Northern California [15], indicating that this upper limit is relevant to healthy populations of individuals living in fluoridated communities in the US. Multiple regression analyses were done to determine the association between plasma fluoride concentrations and blood cell measures contained in the CBC (for details, refer to https://wwwn.cdc.gov/Nchs/Nhanes/2015-2016/CBC_I.htm).

Covariates/ or predictors that were included in the multiple regression analysis were age [16], ethnicity [17] and body mass index (BMI) [18], all of which have been shown to increase WBC counts in children. We also included gender, as women have been shown to have lower leukocyte counts than men [19]. We ran models including the family poverty to income ratio (PIR) as an indicator of socioeconomic status; however, this measure was not statistically significantly associated with changes in WBC counts in any of the models. Dependent variables were blood cell counts available in this subpopulation.

### Statistical analyses

All analyses applied survey weights from the mobile exam center visit (i.e., MEC weights) and the strata and PSU variables to account for the stratified clustered sampling design and to permit generalization to the U. S. population (National Center for Health Statistics, 2013). Descriptive statistics and regression analyses were performed using Stata 17.0 software. Survey-weighted linear regression was used to model blood cell counts as a function of plasma fluoride concentrations while adjusting for covariates (e.g., gender, age and BMI).

## Results

The overall mean and standard deviation water fluoride level for this population was mean of 0.56 ± 0.44 and ranged from 0.07 to 7.32 ppm fluoride. Plasma fluoride levels were a mean of 0.46 ± 0.01 µmolar and ranged from 0.25 to 4.32 µmolar. Gender was 54% male and 46% female, and the mean age was 12.5 ± 4.7 years of age. BMI mean and SD were 22.0 ± 7.5, ranging from 12.3 to 68.6 (see Table 1).

**Table 1:** Descriptive statistics of the subpopulation of children with available plasma fluoride levels

Demographic Characteristic	Subpopulation*
Sex; N(%)	
Female	21,100,607 (54%)
Male	18,032,725 (46%)
Race; N(%)	
Non-Hispanic White	20,782,052 (53%)
Non-Hispanic Black	5,508,195 (14%)
Non-Hispanic Asian	1,702,983 (4.4%)
Hispanic	9,236,116 (24%)
Other/multicategory	1,903,986 (4.9%)
Age; Mean(sd)	12.52 (4.69)
BMI; Mean(sd)	21.98 (7.46)

^*^Subpopulation: weighted values for ages 6 to 19 (inclusive), fluoride plasma comment code = 0, and plasma fluoride less than or equal to 5

^*^Raw *N* = 3,491; weighted *N* = 39,133,332

The results of our regression analyses, including coefficients, 95% upper and lower confidence intervals (brackets) and the p-values for each predictor (plasma fluoride, age, ethnicity, gender and BMI) showed that plasma fluoride was significantly positively associated with water fluoride concentrations (*p* < 0.001), and WBC counts (*p* = 0.014). Among the different types of white blood cells, neutrophils (neutro) (*p* = 0.028) and monocytes (mono) (*p* = 0.006) were significantly positively associated with plasma fluoride concentrations, whereas lymphocytes (lymph), eosinophils (eosino) and basophils (baso) were not (see Table 2).

**Table 2:** Regression analyses of WBCs adjusted for plasma fluoride, age, gender, ethnicity, and BMI*

	Plasma F	Age	Female	Ethnicity (Black)	Ethnicity (Hispanic)	Ethnicity (Asian)	Ethnicity (Other/Mulit)	BMI	Constant	N subpop weighted (raw)
Dependent Variable										
WBC	0.482 (0.107, 0.858)	-0.089 (-0.124, -0.053)	0.397 (0.213, 0.581)	-0.879 (-1.023, -0.074)	0.257 (0.063, 0.450)	0.179 (-0.230, 0.588)	0.082 (-0.298, 0.462)	0.094 (0.071, 0.118)	5.838 (5.392, 6.283)	38,715,039 (3,453)
	*p* = 0.014	*p* < 0.000	*p* < 0.000	*p* < 0.000	*p* < 0.011	*p* = 0.378	*p* = 0.462	*p* < 0.000	*p* < 0.000	
Lymph	0.076 (-0.047, 0.198)	-0.072 (-0.081, -0.063)	0.139 (0.075, 0.205)	-0.160 (-0.237, -0.084)	0.045 (-0.05, 0.133)	0.117 (-0.013, 0.246)	-0.236 (-0.164, 0.117)	0.017 (0.022, 0.021)	2.952 (2.770, 3.133)	38,659,063 (3,447)
	*p* = 0.217	*p* < 0.000	*p* < 0.000	*p* < 0.000	*p* = 0.365	*p* = 0.075	*p* = 0.735	*p* < 0.001	*p* < 0.000	
Neutro	0.364 (0.042, 0.685)	-0.012 (-0.030, 0.028)	0.306 (0.173, 0.440)	-0.684 (-0.780, -0.588)	0.202 (0.084, 0.320)	0.079 (-0.209, 0.388)	0.097 (-0.194, 0.389)	0.070 (0.051, 0.089)	1.994 (1.672, 2.317)	38,659,063 (3,447)
	*p* = 0.028	*p* < 0.936	*p* < 0.000	*p* < 0.000	*p* = 0.002	*p* = 0.581	*p* = 0.501	*p* < 0.000	*p* < 0.000	
Mono	0.041 (0.011, 0.072)	-0.004 (-0.007, -0.002)	-0.006 (-0.020, 0.008)	-0.52 (-0.072, -0.31)	-0.011 (-0.29, 0.007)	-0.39 (-0.72, -0.031)	-0.005 (-0.037, 0.027)	0.006 (0.005, 0.008)	0.482 (0.441, 0.524)	38,659,063 (3,447)
	*p* = 0.009	*p* < 0.001	*p* = 0.366	*p* < 0.000	*p* = 0.214	*p* = 0.027	*p* = 0.748	*p* < 0.000	*p* < 0.000	
Eosino	-0.0002 (-0.021, 0.021)	-0.010 (-0.013, -0.007)	-0.037 (-0.054, -0.020)	0.023 (-0.006, .053)	0.026 (-0.005, 0.058)	0.024 (-0.019, 0.066)	0.016 (-0.023, 0.056)	0.001 (-0.001, 0 .002)	0.363 (0.312, 0.414)	38,659,063 (3,447)
	*p* = 0.981	*p* < 0.000	*p* < 0.000	*p* = 0.113	*p* = 0.100	*P* = 0.263	*p* = 0.860	*p* < 0.001	*p* < 0.001	
Baso	0.003 (-0.007, 0.012)	-0.001 (-0.002, 0.007)	-0.002 (-0.006, 0.003)	-0.001 (-0.008, 0.005)	0.002 (-0.005, 0.009)	0.003 (-0.003, 0.01)	-0.001 (-0.011, 0.008)	.0007 (0.000, 0.001	0.039 (0.031, 0.047)	38,659,063 (3,447)
	*p* = 0.558	*p* < 0.000	*p* = 0.514	*p* = 0.070	*p* = 0.556	*p* = 0.284	*p* = 0.753	*p* = 0.003	*p* < 0.001	
Water F	0.296 (0.167, 0.426)	-0.003 (0.010, 0.005)	-0.003 (-0.031, 0.025)	0.106 (0.026, 0.187)	0.091 (0.001, 0.181)	0.03 (-0.034, 0.093)	0.003 (-0.075, 0.080)	.002 (-0.003, 0.008)	0.370 (0.218, 0.523)	37,854,276 (3,396)
	*p* < 0.000	*p* = 0.443	*p* = 0.816	*p* = 0.011	*p* = 0.048	*p* = 0.347	*p* = 0.07	*p* = 0.405	*p* < 0.000	

^*^Confidence levels, 95% upper and lower confidence intervals (brackets) and the *p* value are listed for each set of independent and dependent variables tested in the regression model using weighted values. Significant values (*p* < 0.05) are highlighted

^*^Ethnicity = white is the reference group; the omnibus test of ethnicity is statistically significant for WBC, Lymph, Neutro and Mono

All other CBC measures (hemoglobin, hematocrit, mean cell hemoglobin concentration, red cell distribution width, platelet count and mean platelet volume) were not significantly associated with plasma fluoride concentrations and are not included in the table.

## Discussion

The CBC with 5-part differential, includes red blood cell count, red blood cell distribution width, and mean cell volume, white blood cell count, platelet count and mean platelet volume, measures of hemoglobin and hematocrit, and sorts the white blood cells into subtypes. White blood cells are recruited by inflammatory cytokines in both acute and chronic inflammation, and are a reliable marker of inflammation [13]. Our finding of a positive association between WBC counts in children aged 6–19 and plasma fluoride concentrations therefore suggests an association between fluoride exposure and increased inflammation.

Among the different subtypes of white blood cells, neutrophils, monocytes, and lymphocytes respond to peripheral inflammation, while eosinphils and basophil are most associated with inflammation related to allergic responses [20, 21]. Lymphocytes produce antibodies and direct cell mediated killing of virus infected and tumor cells [22]. Neutrophils represent about 70% of all white blood cells, and as they enter the blood stream, neutrophils are recruited to sites of tissue damage [23–25], where they are then subsequently cleared by monocytes. Our finding of a significant postive associations between plasma fluoride and both neutrophils and monocytes suggests an effect of fluoride on tissue specific inflammatory changes.

The small intestine is one such possible site. In rats, ingestion of fluoride in drinking water results in inflammatory lesions in the small intestine that appear similar to those found in Crohn’s disease [9, 26, 27], an inflammatory disease most frequently found in the small intestine. Increased neutrophils [28] and moncytes [29]^.^are key players in the chronic inflammation of Crohn’s disease patients. Chronic kidney disease (CKD) in adults is also associated with increased monocyte and neutrophils. Consistent with a relationship between fluoride and kidney function, a previous report using this same NHANES survey data showed a significant association between increased plasma fluoride and a decreased glomerular filtration rate [30], a marker of reduced kidney function [31].

Though we adjusted for gender in our regression analysis, previous analyses of NHANES data sets show that males aged 6 to 19 have relatively higher plasma fluoride levels relative to water fluoride concentrations [12]. This may be due to differences between males and females in fluoride absorption by the kidneys [32] and suggests the possibility that that the effects of water fluoridation are influenced by relatively higher plasma fluoride levels in males.

To determine whether the effects of fluoride on WBC counts might be influenced by other environmental contaminants, such as arsenic, which is known to be a major contaminant of drinking water, we assessed the interaction between fluoride and arsenic in drinking water. We found that while arsenic was associated with changes in hemoglobin, interactions with fluoride were nonsignificant (data not shown).

This study was limited by measures included in the NHANES data sets. This meant that the study was limited to children aged 6 to 19 in the 2013–2014 and 2015–2016 data sets where plasma fluoride analysis was included. Systemic inflammation increases with age and is a risk factor for multiple health effects [33], so further studies to assess the effect of fluoride relative to age in adults are warranted.

Other limitations of this study are the cross-sectional study design, which precludes the inference of causality. However, our finding that fluoride was associated specifically with the WBC subtypes of neutrophils and monocytes, while not affecting lymphocytes, eosinophils and basophils, indicates a specific effect of fluoride in enhancing inflammation. In animal models, fluoride in drinking water has been shown to increase activation of NF-kB in cells and organs throughout the body, including ameloblasts [34] and liver [35]. NF-kB has a critical role in mediating the inflammatory response [36], and our report of an association between plasma fluoride concentrations and WBC counts in children suggests that findings of fluoride associated inflammation in animal models, may also be relevant to human populations.

## Conclusions

Our findings of an association between plasma fluoride concentrations and increased WBC counts in U.S. children and adolescents suggest that ingested fluoride may be an environmental risk factor for inflammation for this population. Dental fluorosis, a biomarker for fluoride exposure, has continued to increase in the U.S. [37, 38] suggesting increasing population based fluoride exposure. This indicates the need for additional studies to assess the effects of fluoride on markers of inflammation in adults and vulnerable human populations.

## Data Availability

The datasets used for these analyses are publicly available (https://wwwn.cdc.gov/nchs/nhanes/Default.aspx).
